# Supplementary figures and images for: Assigning harvested waterfowl to geographic origin using feather δ2H isoscapes: What is the best analytical approach?
Source: PLoS One. 2023 Jul 10;18(7):e0288262. doi: 10.1371/journal.pone.0288262 (PMC10332603; doi:10.1371/journal.pone.0288262)

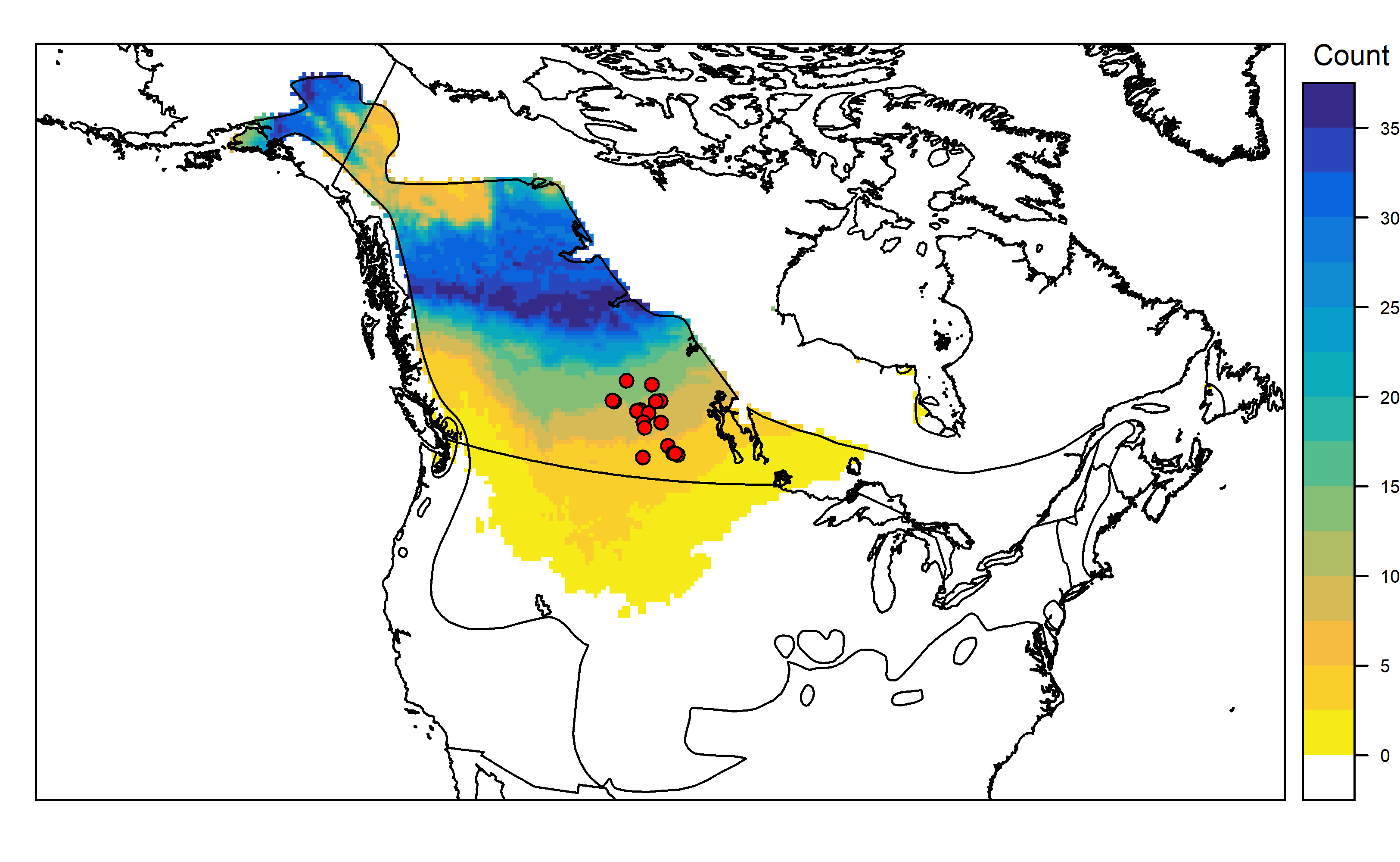

Supplement: S1 Fig — Likely origins of Blue-winged Teal (Spatula discors) harvested in southern Saskatchewan (n = 47, 2014–2018 [9]) using the assignment methods from the original publication (calibration: δ2Hf = -31.6 + 0.93 * δ2Hp; SDresid = 12.8). The colour indicates the number of individuals that were assigned to a given pixel under a 2:1 odds ratio. Harvest locations for samples are shown as red points. (TIF) [file pone.0288262.s001.tif]

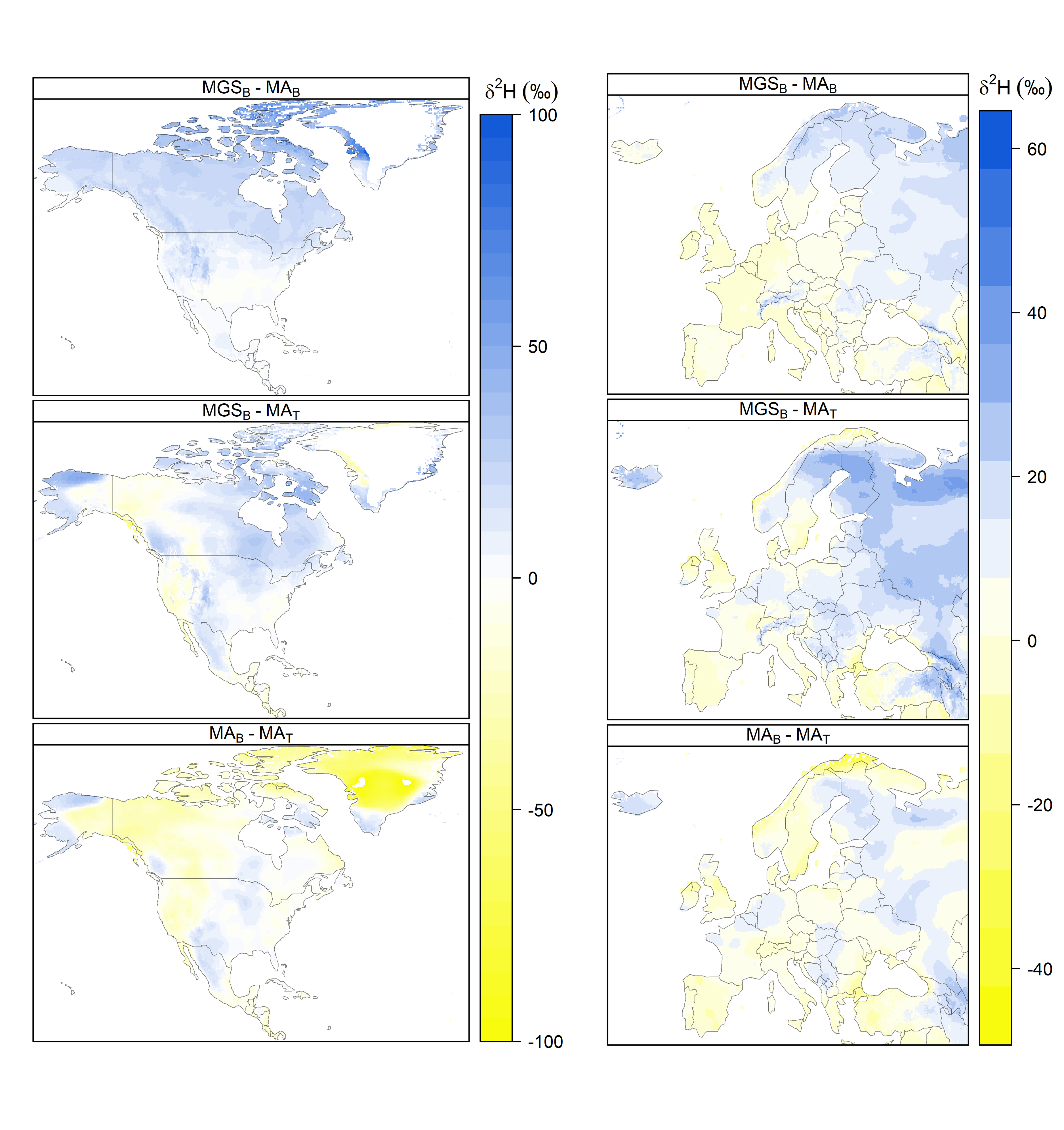

Supplement: S2 Fig — Each panel shows the difference (first isoscape minus second) between paired isoscapes (MGSB−MAB, MGSB−MAT, MAB−MAT): amount-weighted mean growing-season precipitation [50] (MGSB) and amount-weighted mean annual precipitation (MAB, [50]; MAT, [56]). Blue regions represent areas where the first isoscape is much more positive than the second and yellow regions represent areas where the first isoscape is more negative than the second. (TIF) [file pone.0288262.s002.tif]
