# Supplementary material for: Assigning harvested waterfowl to geographic origin using feather δ2H isoscapes: What is the best analytical approach?
Source: PLoS One. 2023 Jul 10;18(7):e0288262. doi: 10.1371/journal.pone.0288262 (PMC10332603; doi:10.1371/journal.pone.0288262)
Supplement: Attachment — Submitted filename: Response to Reviewers.docx [file pone.0288262.s004.docx]

**PONE-D-23-08212**

**Assigning harvested waterfowl to geographic origin using feather *δ*^2^H isoscapes: What is the best analytical approach?**

**Reviewer #1:** Analyses in this paper are well presented and the results well supported. There is some confusion in numbering of figures – apparently what had been Fig S1 in Supporting Information has been moved to the main text, without correcting the numbering of the figures between the main text and Supporting Information (see P 36). There are also some column alignment problems with Table S1. Below are some specific comments to clarify the text.

**Response:** Thank you for the comments! The column alignment in S1 Table has been made consistent (left justified). All other comments are addressed below. We also address the issues with figure number below.

**1. L 26.** For the general reader, please use a phrase that explains more clearly what "amount weighted" means. Amount of what?

**Response:** For the abstract, this description would be too detailed. This is now explained in the Methods on Line 158. In the isoscape literature, this is a common term referring to isotopic averaging based on monthly precipitation collections.

**2. L 36-37. L 36-37.** I suggest "data for individual dabbling duck species"

**Response.** Done (Line 37). But the word “species” was changed to ‘studies’ as these are not species specific in all cases.

**3. L 35-37.** What about diving ducks? As they were also included in the study, the reader expects some analogous comments.

**Response:** Good point – ‘dabbling duck’ was changed to ‘foraging-guild-specific datasets’ (Line 38) to be more inclusive with this statement. It now applies to both dabblers and divers.

**4. L 54.** Replace "that" by "where'

**Response:** Done (Line 55).

**5. L 96-97.** But only if the juveniles are collected before migration. I have seen HY diving ducks that were still molting head feathers upon arrival at wintering areas. Unless you collect the juveniles before they migrate, you will still need to use only flight feathers.

**Response:** It’s a good point that we need to be very clear that feathers are grown at different times and certain feathers groups (e.g., body feathers) are not reliable indicators of moulting location for waterfowl. For this specific line, we state ‘flight-feather moult’ and ‘these newly-formed feathers’ (Lines 71-72).

**6. L 206.** I presume that "Fig 1" here refers to Fig S1 that is now in Supporting Information, as the caption to Fig S1 is missing from the Supporting Information. Please reconcile the numbering, placement, and captions of your figures.

**Response:** It’s not clear what this specific comment refers to. We followed the format for PLOS, which requires the captions to be placed in-line with the text after the first mention of the figure. Fig 1 on Line 208 refers to Fig 1. For the supplementary figures, we have included them at the end of the manuscript separately. If the confusion stems from a lack of a S1 Fig - there was no Figure S1 in the Supporting Information because we named the supplementary figures/tables sequentially (i.e., S1 Table, S2 Fig, S3 Fig). We now realize the format for the figures should be ‘S2 Fig’ not ‘Fig S2’ and have adjusted accordingly. In looking into this, we did realize that one reference to ‘Fig 2’ on Line 466 should have been ‘S3 Fig’, as it was referenced in the text first. This has now been fixed.

**7. L 218.** Cornell is misspelled.

**Response:** Fixed (Line 220).

**8. L 514.** I suggest inserting "choice" after "This".

**Response:** Done (Line 518).

**9. L 529.** Remove the comma after "source" and reinsert after "uncommon", or else remove both commas around this phrase

**Response:** Done. Moved the comma to after ‘uncommon’ (Line 533).

**10. L 540.** Delete the comma after "relationships"

**Response:** Done.

**11. L 550.** Replace “this” by “the Dabbler dataset”. I recommend avoiding use of “this” as a noun, as it is often unclear what “this” refers to.

**Response:** Done (Line 560-561).

**Reviewer #2:** Kusack et al. evaluate the relationship between feather and precipitation hydrogen isotope values for known-origin waterfowl. They used both previously published data from several species, as well as new data collected specifically for the present study. Their goal was to assess which isoscape (mean annual or growing season) yielded the most precise and accurate geographic assignments for different groups (dabbling vs diving) of ducks, with the goal of using this information to improve assignments of unknown-origin waterfowl to their most likely molting locations. The results indicate that mean annual and growing season isoscapes are equally suitable for application to waterfowl (regardless of foraging strategy) and that there were only small differences among the performance of the know-origin datasets. The methods used are generally reliable and the interpretations sound, though I offer a number of specific suggestions/comments below to help improve the manuscript.

My one big-picture comment is related to which calibration equation(s) listed in Table 3 the authors recommend future studies use. That isn’t entirely clear/explicit. For example, if I am studying Mallards should I use van Dijk or the combined dabblers dataset? Relatedly, if I am working on a species of waterfowl other than those presented in this manuscript, should I take the time/effort/expense in developing a calibration equation for my own species or would the dabbling or diving duck datasets be sufficient? More guidance for readers who are interested in using the results of this study for their own species/system would be helpful.

**Response:** Thank you for your detailed review and for the constructive comments. We’ve tried to simplify our recommendations and provide more detailed responses below.

**Specific comments**

**Lines 25-27:** The wording here is unclear. I think the authors are intending to say that calibrations for most aquatic and semi-aquatic species are also currently done using amount-weighted mean growing-season d2H values, but the calibration relationship may not be not as clear for them as for terrestrial-foraging species. However, that isn’t what this sentence says. Perhaps delete everything before the first comma to fix.
**Response:** We’ve removed everything before the comma, as suggested (Line 25).

**Line 30:** Perhaps indicate that 3 of the 4 datasets were previously published and one was collected as part of the current study.
**Response:** Done (Line 30-31).

**Lines: 37-38:** What dataset do the authors recommend be used for diving ducks?
**Response:** Thank you for pointing this out. This was pointed out by both reviewers. We have now modified the wording to be inclusive for both dabbling and diving ducks (Line 38), as we recommend the combined datasets in both cases.

**Line 39:** What “manner” are the authors referring to?
**Response:** Deleted wording.

**Line 55:** What “information” are the authors referring to?
**Response:** Changed information to “assignment method” (Line 56).

**Line 57:** Please add citations to back up this claim.
**Response:** We have modified this statement to include references for migratory connectivity studies that connect breeding and harvest areas (Line 57-58). While these studies make suggestions for management and the obvious benefits, there are no obvious examples of this information informing management decisions to date. This has been made clearer by changing the wording to ‘has the potential to contribute considerably to waterfowl management’ (Line 58).

**Line 73:** “isotopic source and routing” is jargony. Consider deleting everything after the comma in this sentence.

**Response:** These terms are well established in the isotope literature and so we have opted to keep them here.

**Lines 81-82:** Consider changing the order lepidopterans to a common name (butterflies and moths) to match the rest of the list.
**Response:** Done (Line 85).

**Line 115:** Why is this important?
**Response:** Added wording here to clarify that we meant – “When utilizing precipitation isoscapes to assign individuals to origin, it is important” (Line 105).

**Line 118:** Please remove the second “Table”.

**Response:** Done.

**Line 121:** What “relationship” are the authors referring to? Similarly, the relationship is “less clear” than what?

**Response:** Switched the word relationship to ‘calibration’ to be more consistent here (Line 112). The ‘less clear’ is referring to terrestrial species.

**Line 124:** I see >1 calibration relationship in Table 1 that isn’t based on MGS.
**Response:** In Table 1, both waterfowl and non-waterfowl calibrations are provided, but this statement is for waterfowl alone. In Table 1 we also included one calibration (Scaly-sided Merganser [43]) based on surface water measurements (i.e., river δ^2^H), although this is a unique example. The purpose of this statement was to highlight that most waterfowl studies use growing-season values. To avoid confusion, we removed the scaly-sided merganser example from the table and added [43] to the examples of studies that directly measure surface water on Line 116-117. This equation is still in the Supplementary Table for interested readers.

**Line 125:** Need to adjust the wording here. There are obviously many studies that have measured d2H values of surface water. There are not many studies (as I believe the authors intend to indicate) that have measured d2H values of surface water for the purpose of comparison with d2H of feathers from known-origin individuals.
**Response:** Agreed. Slightly modified the language to be clearer that some studies look at the relationship between surface water measurements and duck tissues (Line 116-117).

**Line 136:** This is the first mention of foraging guilds of ducks in the paper outside of the abstract. The audience should know why you’re interested in exploring the calibration relationship for dabbling/diving ducks prior to the end of the introduction where you are stating your research questions and goals.
**Response:** Thank you for the suggestion – we have restructured the introduction to introduce the foraging guilds earlier (Line 90-97) and streamline the flow of the overall introduction.

**Lines 137-141:** This may be more useful earlier on in the introduction to better introduce the importance of understanding calibration relationships for the foraging guilds of ducks.
**Response:** See comment above.

**Line 144:** “underrepresented” relative to what?
**Response:** Underrepresented relative to western North America and Europe, but we’ve switched the word to ‘unrepresented’ (Line 147) as no sampling has occurred in eastern North America (for these purposes).

**Line 158:** What does “largely” mean in this context?
**Response:** Largely in this context refers to the fact that most of the data is from GNIP. We’ve restructured this sentence (Line 161-162) to be clearer.

**Lines 161-162:** Should “MGS-T” instead be “MA-T”?
**Response:** Yes! Good catch! Fixed (Line 165).

**Line 165:** Do the MGS-B and MA-B isoscapes cover the period of 1960-1999? If so, perhaps say that earlier in the text to help the reader understand.
**Response:** Because these data are always expanding, it’s not clear what years are included in the Bowen surface. We believe that they do cover the 1960-1999 period, but cannot verify this. Instead, we provide the citation for the data from the IAEA (IAEA 2015) (Line 164), which is given on the waterisotopes website. At this point, we are more comfortable giving the citation without specifying exactly what years are included here.

**Lines 166-167:** Why did you include these additional predictors in addition to the spatial correlates, and are these predictors significant to model performance?
**Response:** We did not model these isoscape surfaces – they are available as preprocessed surfaces. But, to answer the question, these environmental and geospatial correlates are very important predictors for these models.

**Readme.text file:** The file indicates if the data are or aren’t available for download, but it isn’t clear to me where the data that are available can be obtained. Please make the data available in association with this publication or at least indicate where readers can download the data.
**Response:** At the time of submission the data were not made available. They have now been stored in a Dryad repository (Kusack et al. (in review) – Data from: Assigning harvested waterfowl to geographic origin using feather δ2H isoscapes: What is the best analytical approach? <https://doi.org/10.5061/dryad.9w0vt4bmd>). These data will not be released until the review process is complete – but you can access the files here (<https://datadryad.org/stash/share/Cmr5RzqXGAM-KbGw79_HUMAAswQJfkd_xYUiZJwoG88>). We also added the Dryad reference to the manuscript directly on Lines 538 (Note this citation will need to be updated).

**Table 2:** Does “n” refer to the number of birds or feathers?
**Response:** Both, but most importantly these are individual birds.

**Line 218:** Please change “Cornel” to “Cornell”
**Response:** Done (Line 220).

**Lines 237-239:** Good, but were the sample standards used? And were the same d2H values for those standards used, given that their values have changed, as indicated in Soto et al. 2017? If not, reference 63 (Magozzi et al.) provides an approach to deal with this issue.
**Response:** The same standards were not used in all cases, but that doesn’t matter here as all standards were co-analyzed. As Magozzi et al. describes, it’s more important that the standards were all calibrated on the sample ‘reference scale’. We avoid using this term in the manuscript to avoid confusion with other uses of scale. In our case, we were using standards from Environment Canada, using the values from the co-analysis. Both Clark et al. and Hebert et al. used the original standards (CFS and CHS). van Dijk et al. used the CBS (-198.4 ‰) and KHS (-55.8 ‰) standards in one year and BWB-II (-109.7 ‰) and CHS (-187.9 ‰) in the other year. We used CBS (-198.4 ‰) and KHS (-55.8 ‰). We’ve added the following text on Lines 239-243: “All published data used in our study were processed using the same comparative equilibration methods [62], using the same standards as [62] (i.e., CFS, CHS, BWB) or used standards that have been calibrated relative to the standards in [62] (i.e., KHS, CBS), and therefore should be comparable without any additional transformations [63].”

**Lines 265-267:** It isn’t clear to me why these particular locations are specified here if they are already outside the bounds of North America and Europe.
**Response:** If you use a bounding box (i.e., latitudinal and longitudinal boundaries) to clip NA and EU, you will include some of South America and Africa (respectively) depending on the projection. They could be left in, but we chose to remove them, which is why they are mentioned here.

**Line 279:** Please include a citation for the isocat R package.
**Response:** It is already cited through the methods, but we didn’t explicitly mention the package. We have included specific mention of the isocat package and functions on Line 276-277.

**Lines 280-281 and 285-290:** Why was the odds ratio approach used? Campbell et al. 2020 showed that when known-origin individuals are available (or even when they aren’t), there are other approaches for assignment that have better accuracy/precision than the odds ratio.
**Response:** The odds ratio method, as presented in Campbell et al. 2020 isn’t exactly the same as we have applied it. In Campbell, the probability surface is transformed into odds ratios (i.e., ratios of ratio), then a threshold is applied. In our case, the odds ratio is just describing the probability threshold that we use to select the region of likely origin from the normalized probability surface. You are right, we could transform the probability surface to odds ratios, quantiles, or cumulative sums, but we would end up with a different accuracy/precision trade-off depending on the threshold used. Because we are comparing across groups with the same mathematical methods, we believe using the odds ratio approach is valid here. Further, the method that showed moderately better accuracy (cumulative sum) increases the run time for a single raster by 30+ mins which would increase run time by 10 days for even the smallest Cross Validation test (Note the code for this isocat function is broken at the moment, but we’ve attempted a quick fix to test the efficacy of using this method).

**Line 281:** “uniform” in what way?
**Response:** Uniform is a statistical term that describes a distribution (spatial in this case) where all outcomes are treated as equally likely. We’ve added a definition on Line 285-286.

**Lines 281-283:** Were the samples that the grouped datasets contained from outside NA and EU, not known-origin? Is that why you’re excluding the other regions? Also, are the grouped datasets referring to the ‘Dabblers’ and ‘Divers’ dataset?
**Response:** To be clear, there were no samples from outside of NA or EU. We’ve modified the wording here to clarify (Line 286). This sentence was trying to explain that individuals from a given continent (either NA or EU) were only assigned within the continent of sampling in cases where individuals from both continents were mixed. Otherwise, a single individual would be assigned to both continents simultaneously which isn’t realistic when we know the continent of origin.

**Line 290:** It isn’t clear to me why a 2:1 odds ratio is “conservative”
**Response:** We consider this conservative for two reasons: (1) because we are selecting a moderately high percentage (66%) of the probability density as opposed to a smaller percentage and (2) because we are treating cells within this region as equally likely (i.e., not comparing the relative probability within this region). In theory, you could select a narrower probability density region and have a more precise estimate but a greater chance of assigning an individual to a region of origin not accurate. In our applications, we generally apply the 2:1 odds ratio as a safe trade-off between precision and accuracy.

**Line 297:** You used the function ‘distance’ as opposed to what function from [56]?
**Response:** The equivalent function (i.e., distanceFromPoints(…)) was used in the other publication, but it was from the raster package. We did all coding in the ‘terra’ framework but followed the same methods as [56]. It’s a simple change but needs to be included for consistency. For interested readers they could check the original publication, but that detail isn’t worth including here in our opinion.

**Line 306:** Please change “subset” to the past tense (“subsetted”).
**Response:** Done (Line 311).

**Line 322:** Odd wording. Possibly reword “because we identified this site…” to something like “due to the site’s location on a cranberry farm”.
**Response:** Done (Line 327-328).

**Line 338:** Model fit appears worse, not improved, for the combined diver dataset compared to the Clark dataset (as lines 481-482 also say). Please clarify.
**Response:** Yes, this is a mistake – thank you for spotting it! The sample sizes here were also incorrect. This line must have been missed when updating the results with the addition of new data. It now reads “where only a few additional samples (n = 33) were added to the Clark dataset, model fit was reduced slightly (Table 3).” (Line 343).

**Table 3:** Please also report sample sizes. Also, please define “MA-T”.
Response: Sample sizes were added and the definition for MAT was made clearer here. (Lines 348)

**Lines 431-433:** It isn’t immediately clear to me why a larger range of d2H values would necessarily lead to a lower fit.
**Response:** We agree with the reviewer and have removed this point entirely from the Discussion.

**Lines 487-489:** Could the error associated with the growing season grid be applied to the MA-T grid? Seems like that would be better than not accounting for interpolation error?
**Response:** Yes, it is possible to pair this error surface with the MAT grid using the methods we used here, but we would not recommend it. This error surface is estimating the interpolation error (for the Bowen surfaces), but the Terzer surface contains additional data, uses different interpolation methods, and includes additional predictors. This isn’t something that we want to promote to users and would instead recommend that they use the relevant isoscape for the given error surface.

**Lines 519-522:** These last two sentences are tagged on to this paragraph and don’t really make sense.
**Response:** We agree that the sentences are disjointed with the flow of the rest of the paragraph, but they are both necessary to keep here. Both sentences explain why we did not use other sources of precipitation isoscape data. We’ve changed the order to emphasize the first sentence at the end of the paragraph. We also changed the wording in the final sentence to explain our justification better but have kept both sentences here (Line 523-526).

**Line 535:** I cannot find this appendix.

**Response:** Our apologies. This was meant to point to S1 Table (Line 539). With the Supplementary Information formatting in the last version, this was unclear. This should be fixed now.

**Lines 545-547:** Please be specific about which equation(s) in Table 3 is being recommended.
**Response:** We have modified the recommendations here to be clearer. We do not see either the MA or MGS Bowen surfaces as superior. As such, we’ve given the equations for both MA and MGS (Line 554-555). But we’ve also clarified that we are recommending the dataset, not just the equation, as these data are available for users to derive their own calibrations (Line 549-559).

**Lines 552-552:** which is/are “the most recent calibration equations” being recommended?
**Response:** See comment above.

**Line 558:** Unless I’m reading Table 3 incorrectly, I believe -71.94 should be -71.75. Or perhaps the table is incorrect.
**Response:** Thank you for catching this – the number was incorrect and has been updated it should have been -71.7 (Line 554). All of the calibration values were checked again to ensure accuracy and we noticed that an older version of Table 3 must have been included by mistake. None of the values change by much, but the decimal places are slightly different due to outliers being removed. We also adjusted the significant digits of all isotope values to be one decimal place, as this is the measurement precision of the mass spectrometer**.** All equations given from other papers were left as is, but our summary values, calibration equations and SDresid values are now only provided to one decimal place.

**Figure S2:** I don’t believe Figure S2 is referenced anywhere in the manuscript. Also, what does “MA-T” indicate?

**Response:** Good catch! Yes, one mention of Fig 2 in the main text should have been for “S2 Fig” (note this is now S3 Fig). This was changed (Line 466). “MA_T_” indicates mean annual surface calculated using the Terzer method. As you caught in a comment above, this has now been defined.
